# Endoplasmic reticulum stress enhances unsaturated fatty acid-induced hepatocyte injury in human pluripotent stem cell-derived hepatic culture

**DOI:** 10.1186/s12876-026-04616-9

**Published:** 2026-01-17

**Authors:** Takuma Araki, Keiko Yokoyama, Kinuyo Ida, Yutaka Inagaki, Akihide Kamiya

**Affiliations:** 1 Support Center of Medical Research and Education, Tokai University School of Medicine, 143 Shimokasuya, Isehara, 259-1193 Kanagawa Japan; 2 Department of Molecular Life Sciences, Tokai University School of Medicine, 143 Shimokasuya, Isehara, 259-1193 Kanagawa Japan; 3 Faculty of Pharmacy Department of Pharmaceutical Sciences, Musashino University, 1-1-20 Shinmachi, Tokyo, Nishi-Tokyo-shi 188-0013 Japan; 4 Center for Matrix Biology and Medicine, Tokai University School of Medicine, 143 Shimokasuya, Isehara, Kanagawa 259-1193 Japan

**Keywords:** Unsaturated fatty acid, Endoplasmic reticulum stress, Inducible pluripotent stem cell, Hepatocyte

## Abstract

**Background:**

The liver is a central organ involved in lipid metabolism. Hepatocytes synthesize fatty acids and triglycerides under conditions of excess energy while decomposing fats by β-oxidation under conditions of energy deficiency. When fatty acid metabolism is impaired due to metabolic syndrome, excessive lipid accumulation in the liver increases the risk of fatty liver disease, chronic hepatitis, and liver cancer. Excessive fat accumulation causes various types of cell damage, such as oxidative stress, which causes lipotoxicity. There is a difference in lipotoxicity between saturated and unsaturated fatty acids among various fatty acids, and saturated fatty acids are more toxic. However, there are still many unknowns about the role of unsaturated fatty acids in steatohepatitis remains unclear.

**Methods:**

In the present study, we evaluated the lipotoxicity of saturated and unsaturated fatty acids in a human induced pluripotent stem (iPS) cell-derived hepatocyte culture system. After inducing the differentiation of hepatic progenitor cells and hepatocytes from human iPS cells, palmitic and oleic acids were added. Tunicamycin and thapsigargin were added to induce endoplasmic reticulum (ER) stress. Cell viability and gene expression were analyzed.

**Results:**

When a saturated fatty acid (palmitic acid) was added to human iPS cell-derived hepatocytes, fatty acids alone induced cell death and expression of ER stress-related factors. However, unsaturated fatty acids (such as oleic acid) alone do not exhibit such activity. When oleic acid and ER stress inducers (tunicamycin and thapsigargin) were added simultaneously, strong induction of cell death was observed. Furthermore, the addition of unsaturated fatty acids and the induction of ER stress strongly suppressed the expression of enzymes in the fatty acid synthesis system. These results indicated that saturated fatty acid accumulation induced ER stress, whereas unsaturated fatty acid accumulation alone did not induce ER stress.

**Conclusion:**

The combination of unsaturated fatty acid accumulation and ER stress induces hepatocyte death, thus indicating the importance of endoplasmic reticulum stress in the process of fatty acid-induced cell death.

**Supplementary Information:**

The online version contains supplementary material available at 10.1186/s12876-026-04616-9.

## Background

Long-term liver damage can result in serious liver diseases such as chronic hepatitis, cirrhosis, and liver cancer. In recent years, with the development of antiviral drugs, the incidence of chronic hepatitis B and C has decreased, whereas that of metabolic dysfunction-associated steatohepatitis (MASH), the hepatic manifestation of metabolic syndrome, has increased. MASH is considered a significant risk factor for liver cancer [1]. The liver is the central organ for lipid metabolism, and hepatocytes regulate the amount of triglycerides in cytoplasmic lipid droplets. Excessive intake of carbohydrates and lipids and lack of exercise induce the accumulation of triglycerides through de novo lipogenesis from acetyl-CoA [2]. Hepatic lipid accumulation causes steatosis and is associated with metabolic dysfunction-associated fatty liver disease (MAFLD). For example, a high-fat diet with 60% energy derived from fat in normal mice experimentally induces hepatic steatosis [3]. However, only approximately 20% of the patients with MAFLD develop MASH. An increase in free fatty acids in the liver causes the accumulation and degeneration of lipid droplets in hepatocytes through the synthesis of triglycerides. Triglycerides have been shown to not be very toxic to hepatocytes, as revealed by the analysis of mice lacking the DGAT1 and DGAT2 genes, which are involved in triglyceride formation [4, 5]. Thus, it is thought that not only the simple accumulation of fatty acids in the liver but also various additional multi-step stimuli are necessary for cytotoxicity to hepatocytes and the onset of MASH, which is called the multi-hit hypothesis [6]. These stimuli include oxidative stress associated with β-oxidation due to the deterioration of insulin resistance, endoplasmic reticulum (ER) stress due to the activation of the Unfolded Protein Response (UPR), and also endotoxins derived from the intestine. For example, an excessive increase in free fatty acids disrupts mitochondrial respiratory oxidation, leading to an increase in reactive oxygen species (ROS). Changes in the intestinal microflora due to diet increase the permeability of endotoxins to the small intestine and stimulate MASH pathology. However, the relationship between these stimuli and stress responses, inflammation, and hepatocyte cell death remains to be fully elucidated.

Among MASH-related stress stimuli, ER stress is closely related to lipid metabolism. The ER is important for calcium homeostasis and protein synthesis, transport, and folding [7]. It is the site of fatty acid metabolism that involves the elongation and desaturation of carbon chains in fatty acid synthesis pathways and plays an important role in the formation of various types of fatty acid profiles. Elovl and stearoyl-CoA desaturase (SCD) are involved in these fatty acid modifications [8, 9]. One of these enzymes, Elovl6, regulates liver lipid accumulation, inflammation, and disease [10, 11]. Furthermore, the ER acts as a cholesterol sensor, and SREBP1 present in the ER binds to SREBP cleavage-activating protein (SCAP) and remains on the membrane when sterol concentration is high. However, when the sterol concentration in the ER decreases, SREBP1 is cleaved and translocated to the nucleus to transcribe lipid and cholesterol metabolic enzymes [12]. The SREBP1 regulatory system in the ER is important for lipid and cholesterol homeostasis. The unfolded protein response adapts to ER stress. The disruption of ER homeostasis, such as the accumulation of misfolded proteins, triggers UPR, which restores ER homeostasis by inducing target gene expression, translational repression, and protein degradation. In mammals, these pathways are controlled by three UPR-related molecules: PERK, ATF6, and IRE1, each of which activates a different pathway to form a stress adaptation system [13]. However, sustained and excessive UPR causes cell death as a self-defense reaction. The UPR is also closely involved in lipid metabolism in the liver; for example, IRE1a induces the synthesis of triglycerides [14], and CHOP is involved in the regulation of β-oxidation [15]. Thus, analyses of ER stress and the UPR are important to reveal the mechanism underlying the relationship between lipid metabolism and MASH progression.

Various culture systems have been used to analyze fatty acid accumulation and lipotoxicity in liver cells. Human primary hepatocytes are mainly isolated from noncancerous specimens obtained from liver cancer surgery or from surplus donor livers during liver transplantation therapy. Therefore, it is possible that a sufficient number of cells cannot always be obtained. In order to solve this problem, a hepatocyte expansion culture system using stem cells can be employed. Several methods have been used to induce the differentiation of functional hepatocytes and non-parenchymal cells from human embryonic stem (ES) and induced pluripotent stem (iPS) cells [16]. In addition, a methodology for the generation of hepatic progenitor cells from human ES and iPS cells has been established, as these progenitor cells can be expanded in vitro for a long time to yield a substantial number of cells for analyses and therapies. These cells are highly proliferative in the progenitor cell state; however, their expression of functional liver genes is relatively low. In contrast, mature hepatic gene expression, including glucose and lipid metabolism, occurs by inducing differentiation through the addition of the extracellular matrix and humoral factors [17, 18]. These human pluripotent cell-derived maturated hepatic cells are suitable for studying lipotoxicity and MASH molecular mechanisms.

For instance, in rat hepatocytes, changes in the cytotoxicity of the saturated fatty acids palmitic acid and oleic acid were analyzed, and it was shown that oleic acid was less toxic than palmitic acid alone or a mixture of palmitic acids [19]. In particular, when palmitic and oleic acids were added simultaneously to human hepatoma cell lines, oleic acid suppressed palmitic acid-induced cytotoxicity [20]. However, the cytotoxicity of oleic acid alone is unknown. The addition of oleic acids induces an inflammatory response in human iPS cell-derived liver tissue cultures [21]. These results suggest that the cytotoxicity of unsaturated fatty acids in liver cells is conflicting, and that hepatoma cell lines might not be suitable for this analysis. In this study, a human iPS cell-derived hepatocyte-like culture system was established to evaluate fatty acid cytotoxicity. We found that oleic acid in combination with ER stress significantly induced hepatocyte death.

## Materials and methods

### Materials

Dulbecco’s modified Eagle’s medium (DMEM) and DMEM/Ham’s F12 medium were purchased from Wako Pure Chemical Industries (Osaka, Japan). Penicillin/streptomycin/L-glutamine (100×), dexamethasone, and nicotinamide were purchased from Sigma-Aldrich (St. Louis, MO, USA). Insulin-transferrin-selenium, non-essential amino acids, and HEPES buffer were purchased from Thermo Fisher Scientific (Waltham, MA, USA). Fetal bovine serum (FBS) was purchased from Nichirei Biosciences (Tokyo, Japan). Hepatocyte growth factor (HGF) and epidermal growth factor (EGF) were purchased from PeproTech (Rocky Hill, NJ, USA). The ATP-competitive inhibitor of ROCK-I and ROCK-II, Y-27632, and a potent inhibitor of TGF-β type I receptor (ALK5-TD), A-83-01, were purchased from Wako Pure Chem. Palmitic acid (P0500-10G) and oleic acid (O1008-1G) were purchased from Sigma-Aldrich. The human iPS line ChiPSC18 was purchased from Takara Bio, Inc. (Shiga, Japan). Apoptosis inhibitor Z-VAD-FMK was purchased from Selleck Chemicals (Houston, TX, USA).

### Culture of human iPS cell-derived hepatic progenitor-like cells

Hepatic progenitor cells (HPCs) were differentiated from a feeder-free human iPSC culture system as described previously [18]. Briefly, cells were expanded and preserved in liquid nitrogen using STEM-CELLBANKER® (Takara Bio, Inc.). HPCs were cultured on dishes coated with laminin 5-1-1 fragment (iMatrix-511; Takara Bio, Inc.). Standard culture medium, which was a 1:1 mixture of hepatic colony-forming unit (H-CFU-C) medium and DMEM with 10% FBS and 10^− 7^ M dexamethasone, was used for expansion. H-CFU-C medium consisted of DMEM/F-12 supplemented with 1× insulin–transferrin–selenium, 10 mM nicotinamide, 2.5 mM HEPES buffer solution, 2× penicillin streptomycin glutamine, and 0.1 mM non-essential amino acids. To induce the expansion of hepatic progenitor cell colonies, 0.25 µM A-83-01, 10 µM Y-27,632, 40 ng/mL recombinant human HGF, and 20 ng/mL recombinant human EGF were used to induce the expansion of hepatic progenitor cell colonies.

To induce mature hepatocyte-like differentiation, HPCs were cultured in an 8:1 mixture of hepatocyte maintenance medium (Takara Bio Inc.) and an EHS-gel Basement Membrane Matrix (Wako Pure Chem.) for six days. These differentiated cells were used as human iPS cell-derived hepatocyte-like cells (hiPS-HLCs).

### Addition of fatty acid in cell culture for the analyses of lipid toxicity

Palmitic acid and oleic acid were added to a bovine serum albumin-free, low-endotoxin (Sigma-Aldrich) solution and then solubilized by sonication. This was used as the stock solution. After maturation of the hiPS-HLCs, the culture medium was replaced with fresh medium. Palmitic and oleic acid stock solutions were then added. To add equal amounts of the solution, the stock and control BSA solutions were mixed according to the concentration and added. Finally, 167–833 µM palmitic acid and 0.23–1.43 mM oleic acid were added to the hiPS-HLC culture. In several experiments, 20 µM Z-VAD-FMK pan caspase inhibitor was added to the hiPS-HLC culture. After 48 h of incubation, the cells were subjected to several analyses. The culture supernatant was collected and lactate dehydrogenase (LDH) activity was analyzed according to the manufacturer’s protocol using the LDH Cytotoxicity Detection Kit (Takara Bio Inc.). For mRNA purification, the cells were lysed using RNAiso Plus (Takara Bio Inc.).

### Cell immunocytochemical analyses and Oil-red-O staining

For cytochemical analysis, cultured cells were fixed with 4% paraformaldehyde for 10 min at room temperature. The cells were then incubated with anti-hepatocyte nuclear factor 4a antibody (sc-6556, Santa Cruz Bio, Dallas, TX, USA) and anti-E-cadherin antibody (ECCD-2, Takara Bio Inc.) for 1 h at room temperature. After washing with PBS was performed, the cells were incubated with an Alexa546-conjugated anti-goat IgG (A11056, Thermo Fisher Scientific) and an Alexa488-conjugated anti-rat IgG (A21208, Thermo Fisher Scientific) for 1 h at room temperature. After washing with PBS, the cells were imaged under a Carl Zeiss Axio Observer Z1 using AxioVision version 4.8 software (Carl Zeiss, Jena, Germany).

Some cells were analyzed using Oil-red-O staining after incubation with 4% paraformaldehyde. The Oil-red-O stock solution consisted of 300 mg Oil-Red-O (576-33002, Wako Pure Chem.) was dissolved in 60 ml 2-propanol. This stock solution was diluted with distilled water at a ratio of 6:4 and used as the working solution. Fixed cells were incubated with the working solution for 20 min at room temperature. After washing with distilled water, the cells were then analyzed under a microscope.

### Gene expression analyses using quantitative RT-PCRalyses using quantitative RT-PCR

Total RNA was extracted using RNAiso Plus (Takara Bio, Inc.). Chloroform was added to the solution and centrifuged, after which the supernatant was separated, and RNA was extracted by ethanol precipitation. First-strand cDNA for quantitative PCR was synthesized from 0.5 µg of RNA using ReverTra Ace qPCR RT Master Mix with gDNA Remover (TOYOBO, Osaka, Japan). Target gene expression was corrected for the TATA-binding protein (TBP). Quantitative analysis of target mRNA was performed using a universal probe library system (Roche Diagnostics, Basel, Switzerland) (Supplementary Table 1).

### Western blot analyses

Nuclear proteins were extracted using NE-PER Nuclear and Cytoplasmic Extraction Reagents (Pierce Bio Inc., Waltham, MA, USA). Cytoplasmic protein concentrations were determined using a bicinchoninic acid (BCA) protein assay kit (Pierce Bio Inc.). Ten micrograms of protein were mixed with SDS sample loading buffer, and the samples were electrophoresed using SuperSep™ Ace 10% gel (Wako Pure Chemical) and transferred to an Immobilon-P membrane (Millipore, Billerica, MS, USA.). The membrane was blocked overnight using blocking solution that contained Block Ace Powder (KAC, Kyoto, Japan) diluted with 5% Tween 20 in Tris-buffered saline (TBS-T), followed by incubation with first antibodies shown in Supplementary Table S2. The membrane was washed with TBS-T and incubated with a secondary horseradish peroxidase (HRP) antibody for 1 h at room temperature. The membranes were washed with TBS-T. The proteins were detected using EzWestLumi Plus detection kit (ATTO CORPORATION, Tokyo, Japan). Sample images were acquired using the FUSION SOLO (Vilber, France).

### Statistical analysis

Microsoft Excel (Microsoft, Redmond, WA, USA) was used to calculate standard deviations (SD), and statistically significant differences between samples were determined using the Student’s two-tailed test. For the analysis of more than three groups, ANOVA and Tukey’s tests were performed using Prism7 (GraphPad Software, SD, CA, USA).

## Results

### The effects of the addition of palmitic and oleic acid in human iPS cell-derived hepatocytic culture

**Fig. 1:**
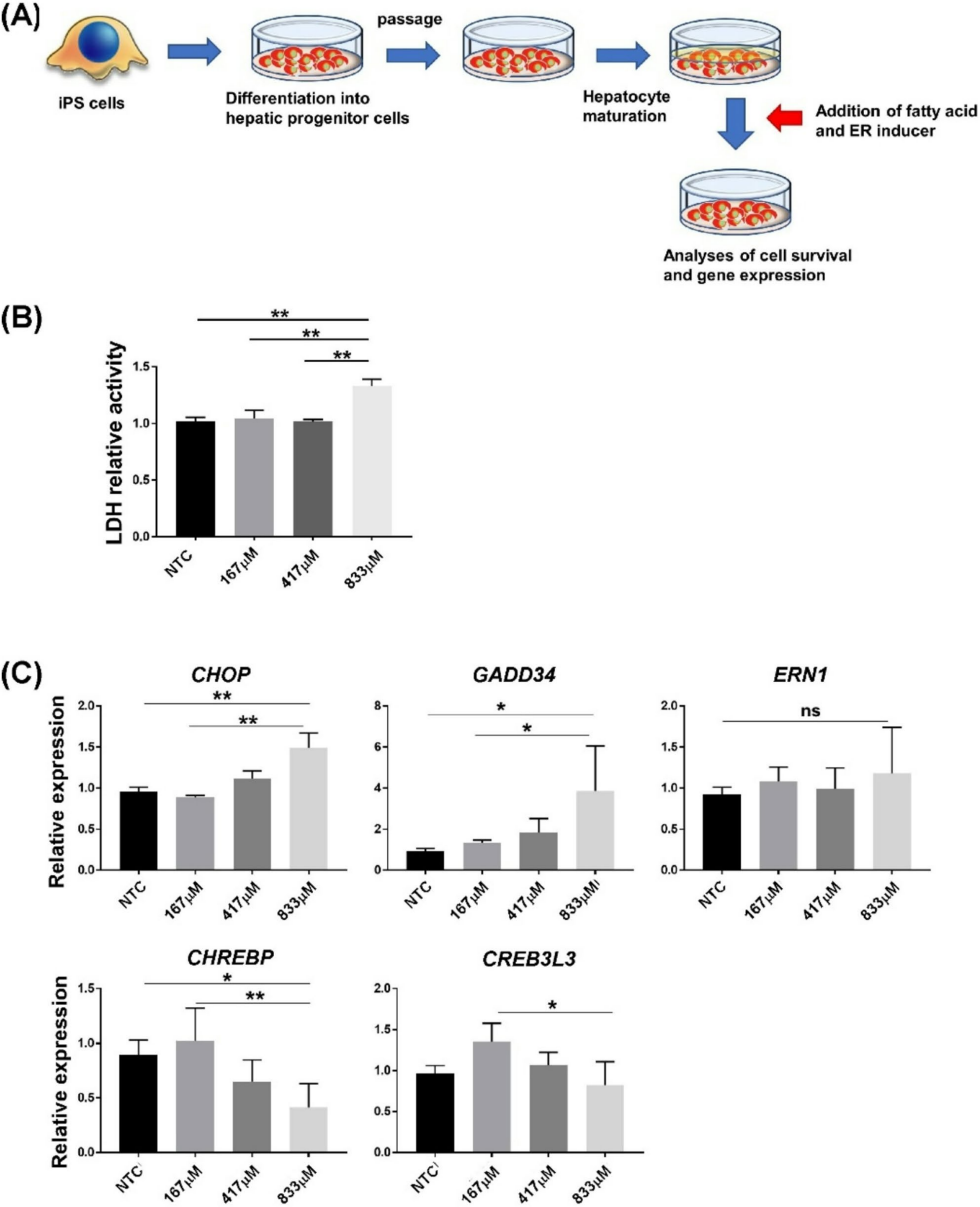
Lipid addition in human iPS cell-derived hepatocyte-like cell (hiPS-HLC) culture. **A** Scheme for the analysis of lipid accumulation using hiPS-HLCs. **B** The LDH assay analyzed cell viability in hiPS-HLCs. Saturated fatty acids (palmitic acid) were added to the cell culture. Results are presented as the mean ± SD (*n* = 4). **C** Expression of ER stress-and lipid metabolism-related genes in hiPS-HLCs treated with palmitic acid. The expression of genes in cells supplemented with control BSA was set at 1.0. Results are represented as the mean expression ± SD (*n* = 4). *P < 0.05, **P < 0.01. ns: not significant

Human iPS cells were induced into the hepatocyte lineage by continuous addition of cytokines and then subcultured in culture dishes coated with laminin fragments to develop hepatic progenitor cells that can be proliferated and cryopreserved for long periods of time [18]. These cells can proliferate for long periods when cultured in the presence of growth factors and acquire mature hepatocyte-like morphology and gene expression by promoting maturation in an extracellular matrix overlay culture. After the induction of maturation, iPS-derived cells expressed HNF4a, a functional hepatocyte marker (Supplementary Fig. 1A). In addition, epithelial cell-cell interactions were also induced. Therefore, we investigated whether fatty acid toxicity in MAFLD and MASH cells could be reproduced using these human iPS cell-derived hepatocytic cell cultures. Fatty acids are classified as saturated fatty acids, such as palmitic acid, and unsaturated fatty acids, such as oleic acid. In addition, saturated fatty acids are more cytotoxic to hepatocytes than unsaturated fatty acids, both in vitro and in vivo. First, we added palmitic acid in a dose-dependent manner to human iPS cell-derived hepatocyte-like cells (hiPS-HLCs) and observed the gene expression and cytotoxicity (Fig. 1A). Palmitic acid-induced cytotoxicity was assessed by measuring the increase in LDH activity (Fig. 1B). In this palmitic acid concentration (833 µM), the cells contained a high density of lipid droplets (Supplementary Fig. 1B). Additionally, high concentrations of palmitic acid suppressed the expression of CHREBP and CREB3L3, which are lipid metabolism-related transcription factors. The ER plays an important role in lipid metabolism and decomposition, and ER stress is known to lead to disruption of lipid metabolic homeostasis [6]. In particular, saturated fatty acids, such as palmitic acid, cause UPR and induce apoptosis in hepatic cell cultures [22]. In an in vivo animal model, saturated fatty acids induced ER stress reactions and liver injury [23]. Thus, in order to analyze the relationship between palmitic acid-induced liver injury and ER stress, we examined UPR activation in this culture system using gene expression. High concentrations of palmitic acid induced the expression of ER stress-related genes such as CHOP and GADD34 (Fig. 1C). These results show that saturated fatty acid accumulation in human iPS-derived hepatic cultures induces both lipotoxicity and UPR signals.

**Fig. 2:**
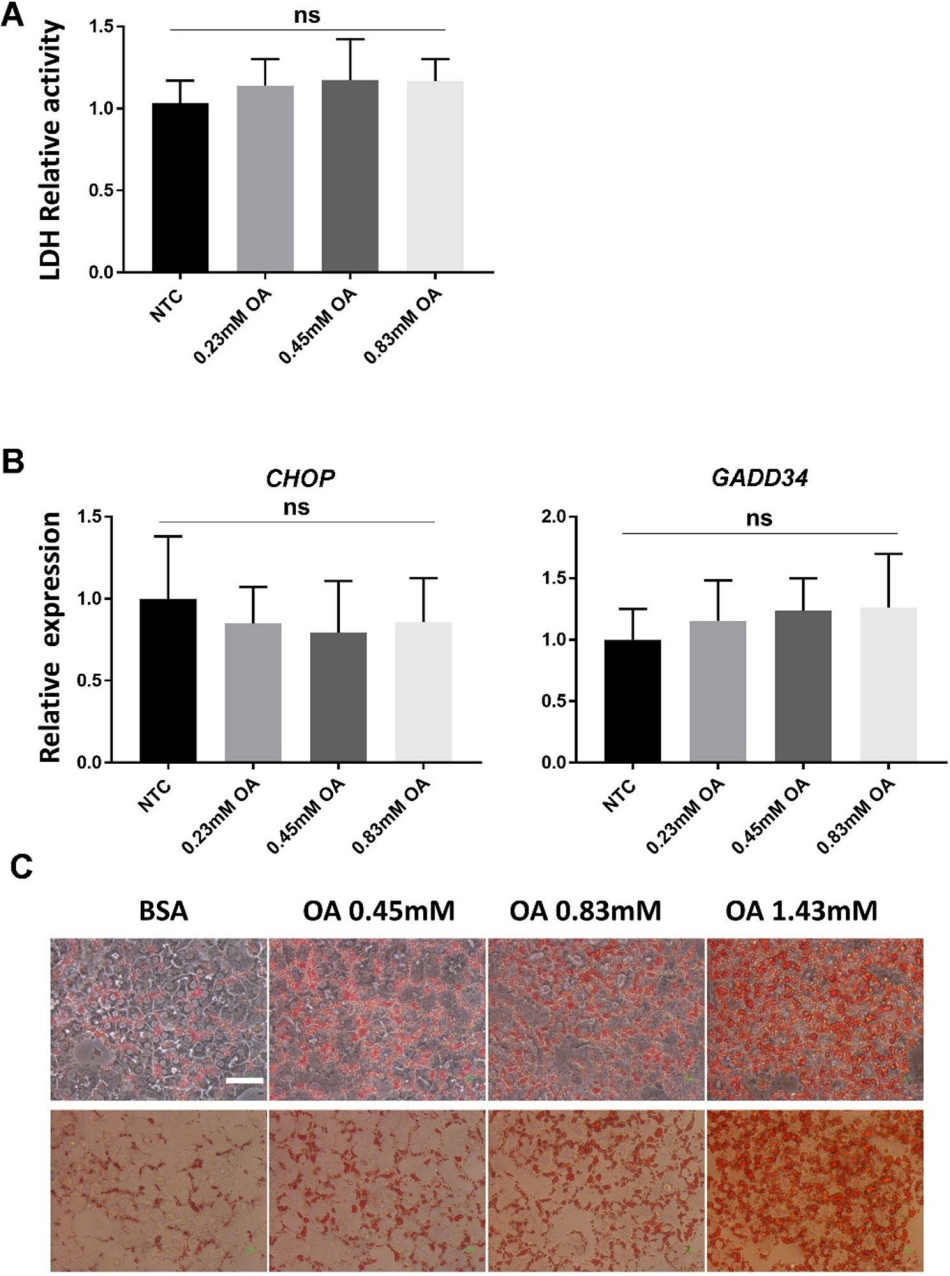
Effect of unsaturated fatty lipids on cell survival and gene expression in hiPS-HLCs. **A** The LDH assay analyzed cell viability in hiPS-HLCs. The unsaturated fatty acid oleic acid (OA) was added to the cell culture. Results are represented as the mean expression ± SD (*n* = 4). ns: not significant. **B** Expression of ER stress-related genes in hiPS-HLCs treated with oleic acid. The expression of genes in cells supplemented with control BSA was set to 1.0. Results are represented as the mean expression ± SD (*n* = 4). ns: not significant. **C** Lipid accumulation in hiPS-HLCs supplemented with oleic acid was analyzed by Oil-red-O staining. White line, 50 μm

In contrast, an increase in LDH activity of hiPS-HLCs was barely detectable in cultures supplemented with unsaturated oleic acid, even at high concentrations (Fig. 2A). Unlike palmitic acid, oleic acid did not induce expression of ER stress genes such as CHOP and GADD34 (Fig. 2B). However, the addition of oleic acid resulted in many lipid droplet-containing cells, inducing excessive lipid accumulation in the cells in a dose-dependent manner (Fig. 2C). These results indicate that the reactivity of saturated and unsaturated fatty acids to human iPS cell-derived hepatocytes was different, and it was possible to construct an in vitro model that reproduces simple fatty liver using human iPS-hep culture by adding unsaturated fatty acids.

### Induction of hepatocytic cell death by unsaturated fatty acids and Endoplasmic reticulum stress

**Fig. 3:**
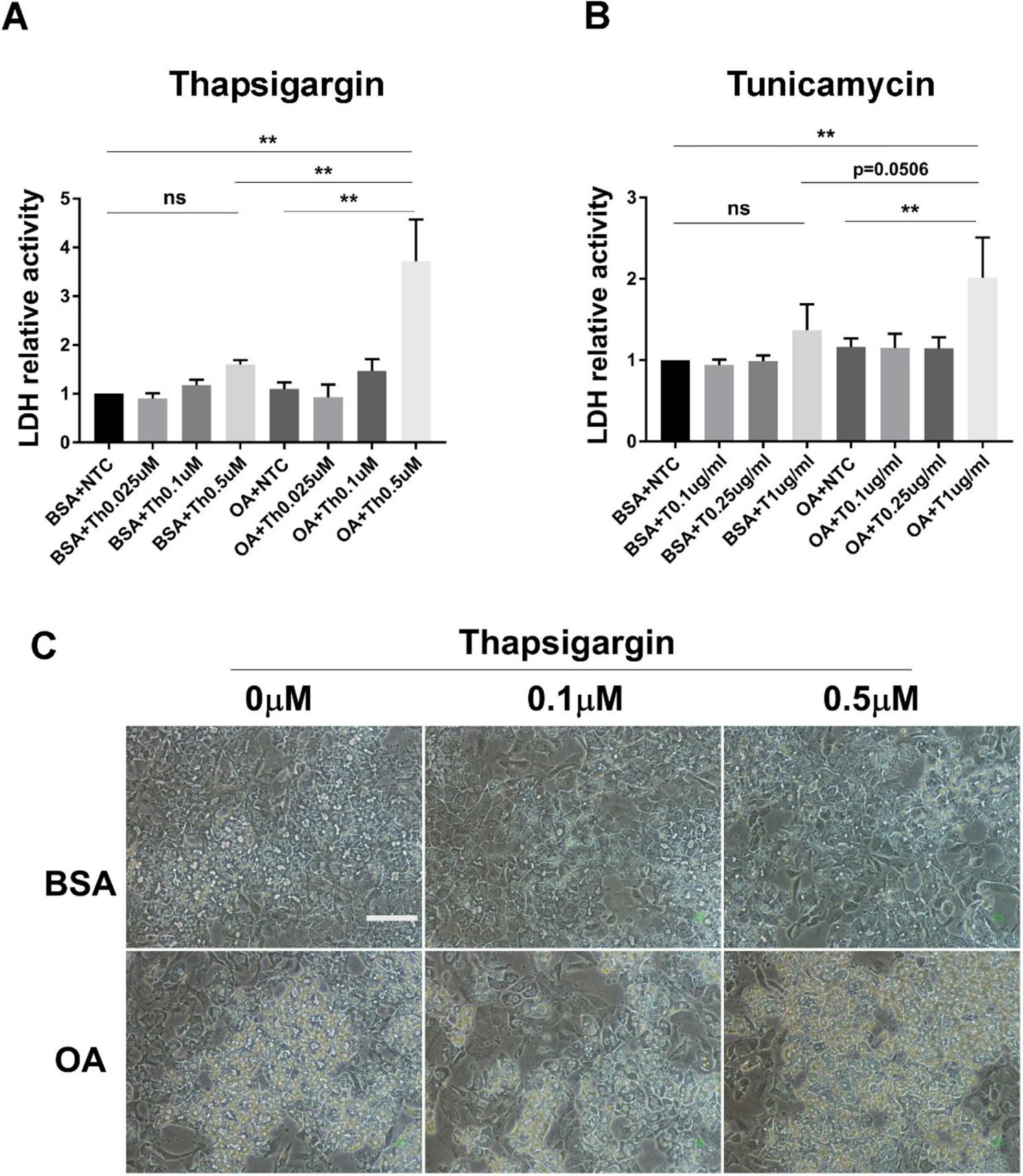
Combination of oleic acid and ER stress inducers caused lipid-accumulated hepatic injury.** A **and** B** Changes in cell viability induced by the combination of ER stress inducers and oleic acid. After 48 h of incubation with thapsigargin (**A**) and tunicamycin (**B**), the LDH activity was analyzed. Results are presented as the mean ± SD (*n* = 3). **P < 0.01. ns: not significant. **C** Cell morphology of hiPS-HLCs stimulated with oleic acid and the ER stress inducer thapsigargin. White line, 100 μm

As shown above, the addition of palmitic acid, but not oleic acid, induced cell injury in human iPS cell-derived hepatic cultures. The progression of liver inflammation during simple liver steatosis requires several stressors or molecular stimulations. Therefore, we investigated factors and stressors that could induce liver inflammation and cell death in combination with oleic acid. As aforementioned, ER stress-related genes were induced by saturated fatty acids, but not by unsaturated fatty acids (Figs. 1C and 2B). Thus, we tried to co-stimulate the ER stress inducers tunicamycin or thapsigargin with unsaturated fatty acids in hiPS-HLCs. In the human hepatic carcinoma cell line HepG2, the induction of endoplasmic reticulum stress by the addition of thapsigargin alone induced cell death (Supplementary Fig. 2A). In contrast, ER stress alone barely induced cell injury, as shown by the LDH activity in hiPS-HLCs (Supplementary Fig. 2B). In particular, neither thapsigargin nor tunicamycin significantly induced cell death in hiPS-HLCs in the absence of oleic acid (Fig. 3A and B, when compared with BSA + NTC vs. BSA + thapsigargin or tunicamycin). Therefore, oleic acid and an ER stress inducer were simultaneously added to the culture medium. Induction of cell death was barely observed in the presence of oleic, with or without low concentrations of ER stress inducers. In contrast, the combination of high concentrations of thapsigargin or tunicamycin, in addition to oleic acid, changed cell morphology and induced cell injury (Fig. 3A-C). We demonstrated that hiPS-HLCs were damaged by the addition of a combination of unsaturated fatty acids and the induction of ER stress.

## Effects of Endoplasmic reticulum stress inducers on adipogenic human iPS cell-derived hepatocytes

**Fig. 4:**
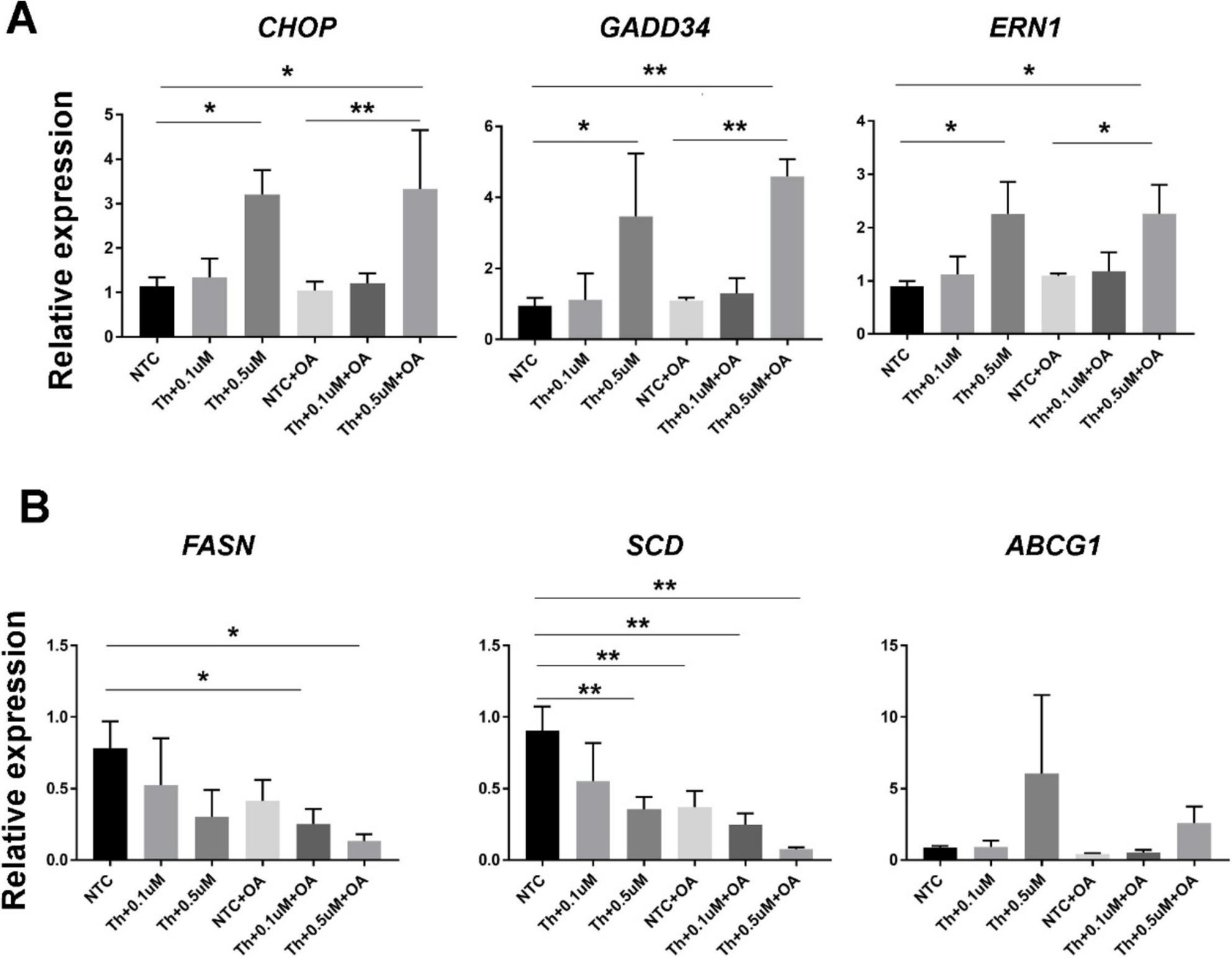
Changes in ER stress marker genes and lipid metabolic genes in hiPS-HLCs supplied with the combination of oleic acid and an ER stress inducer. **A** Expression of ER stress marker genes induced by oleic acid and thapsigargin treatment. **B** Expression of fatty acid synthesis genes induced by oleic acid and thapsigargin treatment. Results are represented as the mean expression ± SD (n = 3). *P < 0.05, **P < 0.01.

The expression of ER stress genes such as CHOP, GADD34, and ERN1 were induced by thapsigargin alone and in combination with oleic acid and thapsigargin in hiPS-HLCs (Fig. 4A). A similar effect was observed following tunicamycin stimulation (Supplementary Fig. 3A). The expression of genes involved in fatty acid synthesis, such as Fasn and SCD, in hiPS-HLCs was suppressed by the addition of oleic acid, and was even more strongly suppressed by the addition of thapsigargin and tunicamycin (Fig. 4B and Supplementary Fig. 3B). Western blot analyses revealed that the combination of OA and thapsigargin induced the protein expression of early UPR markers, specifically BiP (GRP78) and IRE1α, in hiPS-HLCs. In contrast, the levels of other ER-resident chaperones, including Calnexin and protein disulfide isomerase (PDI), were found to be decreased (Supplementary Fig. 4A). Furthermore, stimulation with oleic acid and ER stress inducers led to a downregulation of ACSL1, which is involved in fatty acid activation, and AceCS1 (acetyl-CoA synthetase 1), an enzyme responsible for synthesizing acetyl-CoA (Supplementary Fig. 4B). These results suggest that, although the mechanism that suppresses fatty acid synthesis due to excess fatty acid accumulation is maintained, lipid degradation in the ER is impaired by ER stress inducers, leading to the suppression of liver function and hepatocyte death.

Various molecular mechanisms, including apoptosis and necrosis, are thought to be involved in lipotoxicity-induced cell death. To investigate this, we evaluated the effect of Z-VAD-FMK, a pan-caspase inhibitor capable of blocking apoptosis, on the observed cell death. However, the increase in LDH activity induced by the treatment of hiPS-HLCs with OA and ER stress inducers was not suppressed by the addition of Z-VAD-FMK (Supplementary Fig. 5). These results suggest that molecular mechanisms other than classical apoptosis are likely involved in the hepatocyte death induced by the synergy of OA and ER stress.

## Discussion

Lipotoxicity is the accumulation of excess lipids and metabolic abnormalities that cause hepatocyte degeneration and injury [24]. Most studies indicate that lipotoxicity is associated with several fatty acids, sphingolipids, and phospholipids. Saturated fatty acids, such as palmitic acid, have been reported to strongly induce ER stress and cell death, such as apoptosis and pyroptosis in hepatocytes. In contrast, unsaturated fatty acids such as oleic acid are known to reduce lipotoxicity caused by saturated fatty acids. For example, oleic acid exerts a protective effect against pyroptosis [25]. However, it remains unclear whether unsaturated fatty acids directly induce the progression of liver injury under various stress conditions. In this study, we have analyzed the effects of excessive intake of palmitic acid and oleic acid on human pluripotent stem cell-derived hepatocytes. We previously established a hepatic differentiation culture system from hepatic progenitor cells that can proliferate for a long time and be cryopreserved by continuously adding cytokines to human iPS cells [18]. Palmitic acid induced both cell death and ER stress, whereas oleic acid did not induce cell death or the UPR. Furthermore, cell death, which was not observed with oleic acid alone or with an ER stress inducer alone, was synergistically induced by the addition of both compounds. The ER is an important intracellular organelle involved in fatty acid metabolism, and various transcriptional regulatory mechanisms of fatty acid metabolism are active. When this transcriptional regulatory function is disrupted by ER stress, abnormalities in fatty acid metabolism can occur, which may lead to the induction of cellular lipotoxicity. In addition, the difference seen in UPR signal activation upon the addition of saturated and unsaturated fatty acids may be related to the difference in fatty acid cytotoxicity. In our culture system, palmitic acid alone activated the UPR signal, and there may be a difference in the transcriptional regulators activated by excessive accumulation of fatty acids. Furthermore, we observed that the induction of cell death by oleic acid and ER stress was accompanied by an upregulation of early UPR markers, such as BiP (GRP78) and IRE1α, while the expression of Calnexin and PDI was concurrently reduced. The decrease in these molecular chaperone-related protein levels suggested that the adaptive UPR signaling may have succumbed to decompensation, ultimately triggering cell death. In the future, by analyzing the differences in the molecular mechanisms of such transcriptional regulation, it may be possible to obtain new insights into the distinct roles of saturated and unsaturated fatty acids in cytotoxicity. Elucidating the precise mechanisms by which ER stress and fatty acid metabolism signals intersect to drive cell death remains a critical objective for our future research. Interestingly, cell death was only induced by ER stress in the liver cancer cell line HepG2 cells. This suggests that the lipid and stress responsiveness of living liver cells cannot be reproduced in these cancer cell lines and that human iPS cell-derived liver cells might be useful for such experiments.

When oleic acid was added to human iPS cell-derived hepatocyte cultures, lipid droplets accumulated within these cells, as confirmed by Oil-red-O staining. During this metabolic process, the gene expression of fatty acid metabolic enzymes such as Fasn and SCD was significantly suppressed, indicating that excessive accumulation of fatty acids inhibited de novo fatty acid synthesis. The addition of an ER stress inducer to this process further suppresses Fasn and SCD expression. Therefore, lipid-accumulated hepatocytes might have a defensive response against excess fatty acids by suppressing de novo fatty acid synthesis and promoting β-oxidation. In contrast, the inhibition of the fatty acid metabolic pathway may lead to hepatocellular injury. During the onset of MASH, increased β-oxidation due to excess free amino acids causes mitochondrial abnormalities, thus leading to increased ROS production. This is associated with induction of hepatocellular injury [6]. During the process of cell injury caused by unsaturated fatty acids and ER stress induction in our culture system, the defensive response against fatty acid accumulation may be affected by ER stress induction, possibly inducing cell injury.

## Conclusion

In this study, we demonstrated that oleic acid, an unsaturated fatty acid, does not show strong cytotoxicity on its own, unlike palmitic acid, a saturated fatty acid, which shows cytotoxicity under ER stress. When unsaturated fatty acids are used alone, a MAFLD-like state with excessive intracellular lipids is observed, whereas adding ER stress, which is known to induce MASH, can induce hepatocyte death. This may be applicable for the analysis of the molecular mechanism of the progression from MAFLD to MASH, which remains unknown. 

## Supplementary Information

Supplementary Material 1.

## Data Availability

Data are available from the corresponding author upon reasonable request.

## Abbreviations

EGF: Epidermal growth factor
ER: Endoplasmic reticulum
ES: Embryonic stem
FBS: Fetal bovine serum
HGF: Hepatocyte growth factor
iPS: Induced pluripotent stem
LDH: Lactate dehydrogenase
MAFLD: Metabolic dysfunction-associated fatty liver disease
MASH: Metabolic dysfunction-associated steatohepatitis
SCD: Stearoyl-CoA desaturase
UPR: Unfolded protein response
