# Supplementary material for: Endoplasmic reticulum stress enhances unsaturated fatty acid-induced hepatocyte injury in human pluripotent stem cell-derived hepatic culture
Source: BMC Gastroenterol. 2026 Jan 17;26:118. doi: 10.1186/s12876-026-04616-9 (PMC12895965; doi:10.1186/s12876-026-04616-9)
Supplement: Supplementary file 1 — Supplementary Material 1. [file 12876_2026_4616_MOESM1_ESM.pdf]

Endoplasmic reticulum stress enhances unsaturated fatty acid-induced hepatocyte injury in human pluripotent stem cell-derived hepatic culture

Takuma Araki, Keiko Yokoyama, Kinuyo Ida, Yutaka Inagaki, Akihide Kamiya

Supplementary Figure legends

Supplementary Figure 1 Hepatocyte culture derived from human iPS cells. (A) Differentiation of hiPS-HLCs as evaluated by hepatocyte marker genes. The cells were stained with E-cadherin (green) and HNF4α (red). (B) Lipid accumulation in the hiPS-HLCs supplemented with palmitic acid was analyzed by Oil-red-O staining. White line, 50 μm.

Supplementary Figure 2 Cell injury induced by oleic acid and thapsigargin. (A) Change in HepG2 viability by the combination of the ER stress inducers thapsigargin and oleic acid. (B) Change in hiPS-HLC viability by the combination of the ER stress inducers thapsigargin and oleic acid. Results are presented as the mean (n =2).

Supplementary Figure 3 Expression of ER stress marker genes and lipid metabolic genes in hiPS-HLCs stimulated with oleic acid and tunicamycin. (A) Expression of ER stress marker genes induced by oleic acid and tunicamycin. (B) Expression of fatty acid synthesis-related genes induced by oleic acid and tunicamycin. Results are presented as the mean ± SD (n =3). Significant differences from the NTC group are indicated. *P<0.05, **P<0.01.

Supplementary Figure 4. Expression of ER stress markers and lipid metabolic proteins in hiPS-HLCs stimulated with oleic acid (OA) and thapsigargin (Th). (A) Western blot analysis of the ER stress related proteins BiP (GRP78), IRE1α, PDI, and calnexin following treatment with oleic acid and

thapsigargin. (B) Western blot analysis of the lipid metabolic enzymes ACSL1 and AceCS1 following treatment with oleic acid and thapsigargin.

Supplementary Figure 5. Cell viability in hiPS-HLCs assessed by the LDH assay. The unsaturated fatty acid oleic acid (OA) and an ER stress inducer (Ind) were added to the cell culture. To inhibit apoptosis, Z-VAD-FMK (Z-vad) was added to the culture. Results are presented as the mean ± SD (n = 2-3). The activity in cells treated with OA and the ER stress inducer was set to 1.0. ns, not significant.

Supplementary Table S1
PCR primers for the detection of human gene expression

**Mouse genes**	**Forward primer (5'→3')**	**Reverse primer (5'→3')**	Probe number
TBP	cccatgactcccatgacc	tttacaaccaagattcactgtgg	51
GADD34	gcttctggcagaccgaac	gtagcctgatggggtgctt	24
ERN1	gcgaagcatgtgctcaaac	tctgtcgctcacgtcctg	50
CHREBP	tacgctgcacaactggaagt	ccgttgaaggactcaaacaga	89
CREB3L3	gcatcctgagacacgtgga	tgaggaagtcgtcagagtcg	62
FASN	caggcacacacgatggac	cggagtgaatctgggttgat	11
SCD	cctagaagctgagaaactggtga	acatcatcagcaagccaggt	82
ABCG1	tcagggacctttcctattcg	ttcctttcaggagggtcttgt	22

Supplementary Table S2
First and second antibodies for Western blot analyses

Antigen	Cat number	Supplier	Dilution
BiP	3177	Cell signaling technologies	1/1000
Calnexin	2679	Cell signaling technologies	1/1000
βActin	A5441	Sigma	1/2000
ASCL1	9189	Cell signaling technologies	1/1000
IRE1α	3294	Cell signaling technologies	1/500
AceCS1	3658	Cell signaling technologies	1/1000
PDI	3501	Cell signaling technologies	1/1000
Second Antibody (HRP-conjugated)			
Anti-Rabbit IgG	AP132P	Cell signaling technologies	1/1000
Anti-Mouse IgG	AP124P	Cell signaling technologies	1/1000

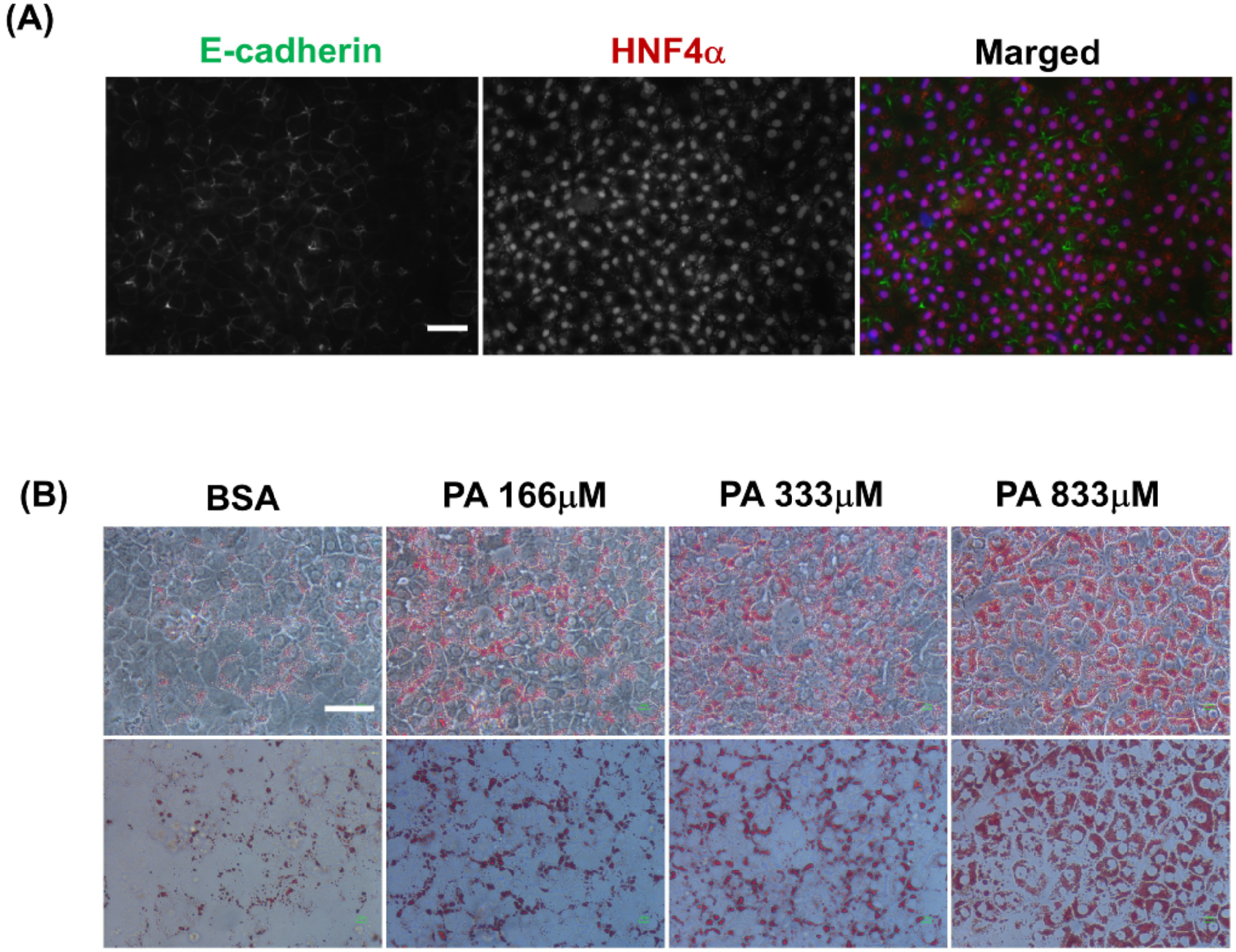

Supplemental Figure 1 Hepatocytic culture derived from human iPS cells. (A) Differentiation of hiPS-HLC evaluated by hepatocyte marker genes. Cells were stained with E-cadhelin (Green) and HNF4α (red). (B) Lipid accumulation in hiPS-HLCs supplemented with palmitic acid was analyzed by Oil-red-O staining. White line, 50 μm.

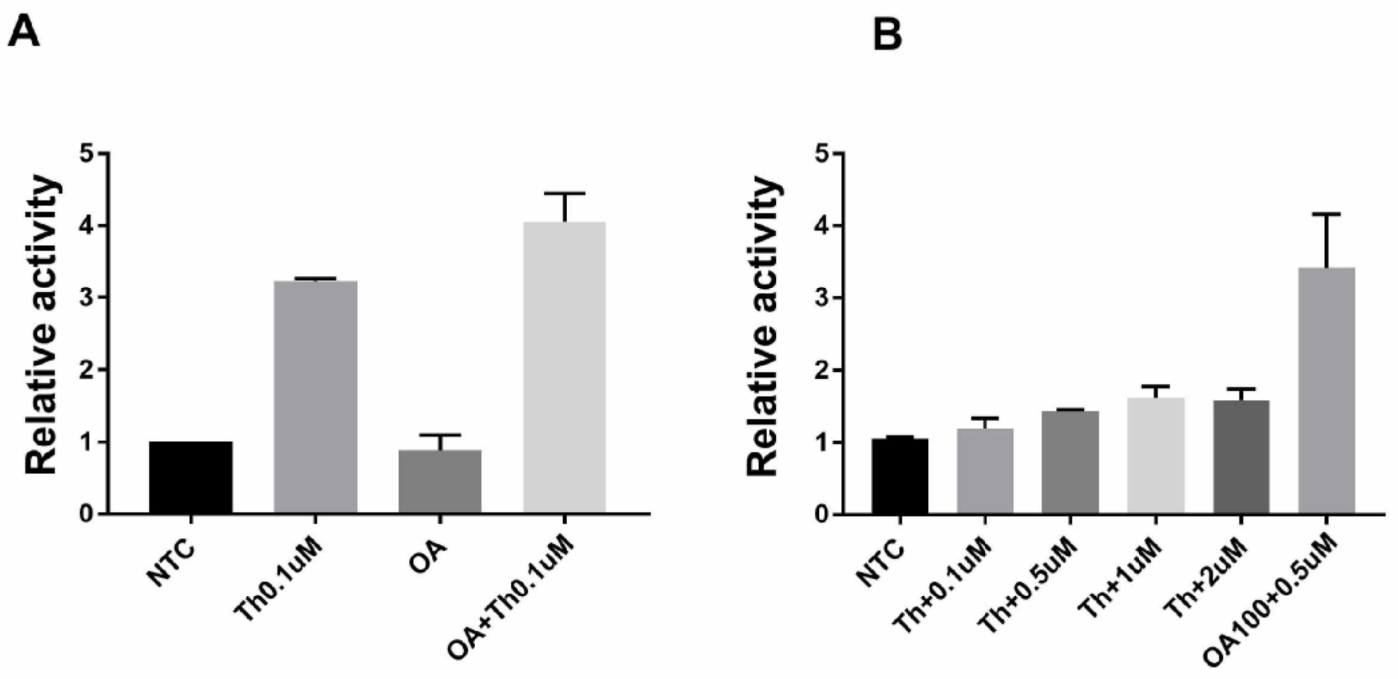

Supplemental Figure 2 Cell injury induced by oleic acid and thapsigargin. (A) Change in HepG2 viability by the combination of the ER stress inducers thapsigargin and oleic acid. (B) Change in hiPS-HLC viability by the combination of the ER stress inducers thapsigargin and oleic acid. Results are presented as the mean (n =2).

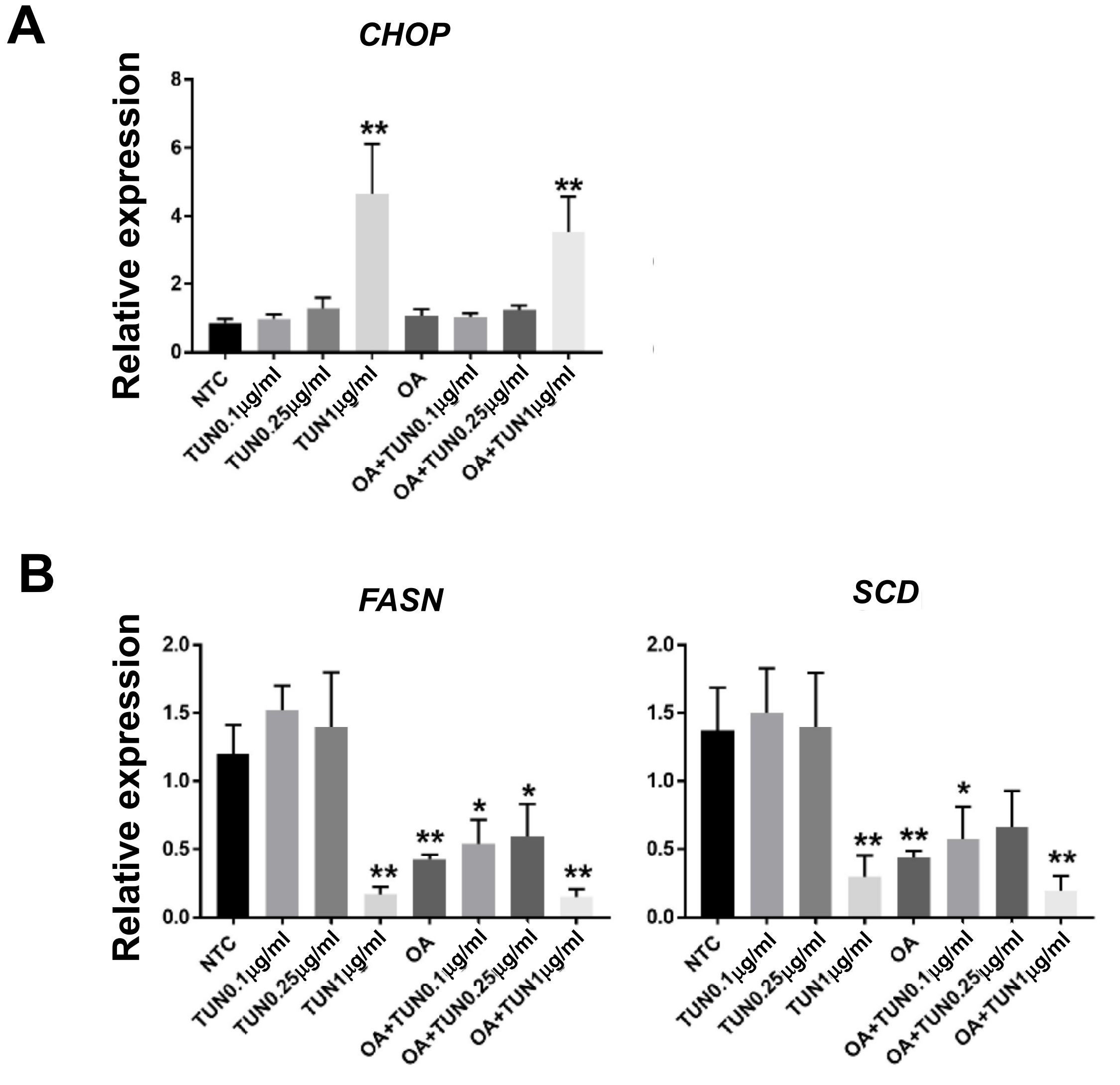

Supplementary Figure 3 Expression of ER stress marker genes and lipid metabolic genes in hiPS-HLCs stimulated with oleic acid and tunicamycin. (A) Expression of ER stress marker gene CHOP induced by oleic acid and tunicamycin. (B) Expression of fatty acid synthesis-related genes induced by oleic acid and tunicamycin. Results are presented as the mean ± SD (n =3). Significant differences from the NTC group are indicated. *P<0.05, **P<0.01.

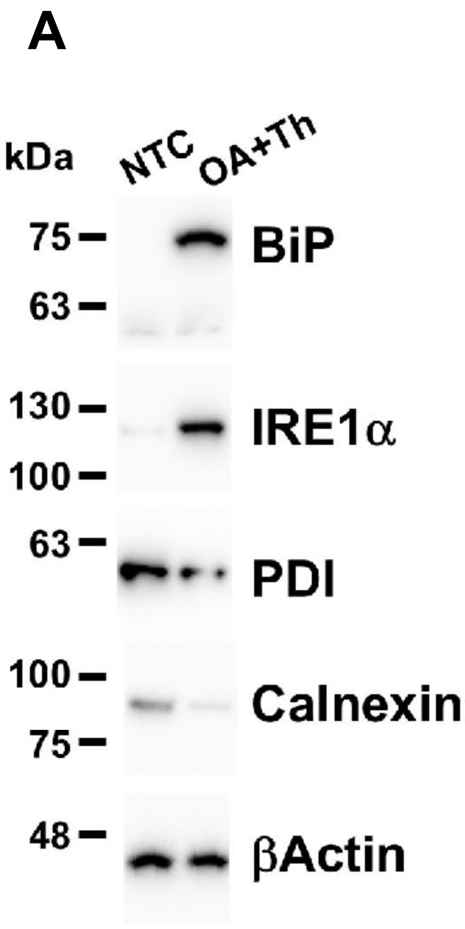

Supplementary Figure 4. Expression of ER stress markers and lipid metabolic proteins in hiPS-HLCs stimulated with oleic acid (OA) and thapsigargin (Th). (A) Western blot analysis of the ER stress related proteins BiP (GRP78), IRE1α, PDI, and calnexin following treatment with oleic acid and thapsigargin. (B) Western blot analysis of the lipid metabolic enzymes ACSL1 and AceCS1 following treatment with oleic acid and thapsigargin.

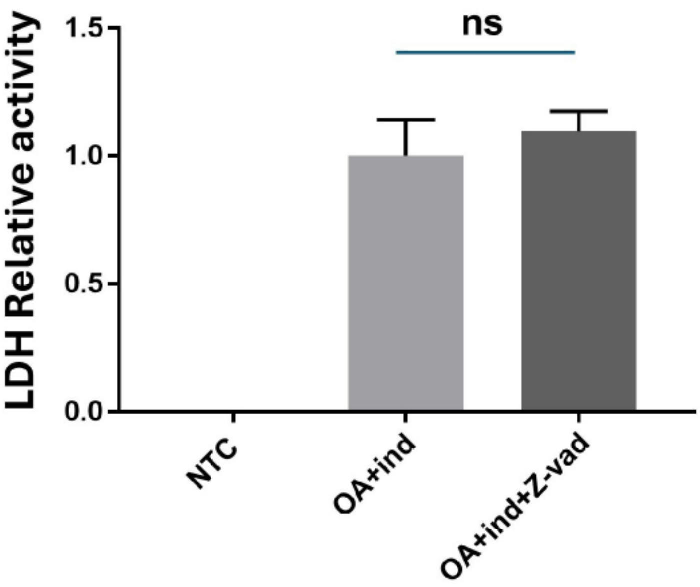

Supplementary Figure 5. Cell viability in hiPS-HLCs assessed by the LDH assay. The unsaturated fatty acid oleic acid (OA) and an ER stress inducer (Ind) were added to the cell culture. To inhibit apoptosis, Z-VAD-FMK (Z-vad) was added to the culture. Results are presented as the mean ± SD (n = 2-3). The activity in cells treated with OA and the ER stress inducer was set to 1.0. ns, not significant.
